# Association between pesticide exposure intensity and self-rated health among greenhouse vegetable farmers in Ningxia, China

**DOI:** 10.1371/journal.pone.0209566

**Published:** 2018-12-21

**Authors:** Jiangping Li, Lijun Dong, Danian Tian, Yu Zhao, Huifang Yang, Xiaoyu Zhi, Lingqin Zhu

**Affiliations:** 1 Department of Epidemiology and Health Statistics, School of Public Health and Management, Ningxia Medical University, Yinchuan, China; 2 Department of Occupational and Environmental Health, School of Public Health and Management, Ningxia Medical University, Yinchuan, China; 3 Department of Hygienic Chemistry, School of Public Health and Management, Ningxia Medical University, Yinchuan, China; 4 Department of infection control, The first people' hospital of Yinchuan, Yinchuan, China; University of Miami, UNITED STATES

## Abstract

**Background:**

Self-rated health (SRH) has been shown to be a stronger comprehensive predictor of health status than the clinical record. Although an association between specific pesticide exposures and health conditions has been reported in different populations, data on the relationship between pesticides exposure intensity (PEI) and SRH in greenhouse farmers is scarce. The aim of the current study was to evaluate this association among vegetable greenhouse farmers in Yinchuan City, western China.

**Methods:**

Three consecutive cross-sectional studies were conducted in the years 2015, 2016 and 2017. Face-to-face interviews by trained investigators, using questionnaires, were performed. PEI was calculated by a validated method and then categorized into high, middle and low groups. SRH was measured via a single ten-point scale question and then divided into excellent (score >5) and poor SRH (score ≤5). A multivariable logistic regression model was used to evaluate the association. Meanwhile, the dose-response and interaction effects were estimated.

**Results:**

A steady association between high PEI and poor SRH (OR: 1.55, 95% CI: 1.05–2.28 in the full model) was identified. Although high PEI was significantly associated with poor SRH in males and the Han ethnicity group, no significant association was found with poor SRH in females or those of Hui ethnicity. Interaction effects of education level and frequency of breakfast with PEI were determined (*P*_interaction_ = 0.04 and 0.02, respectively); synergistic enhanced effects for poor SRH were observed.

**Conclusion:**

These findings indicate that high PEI might be associated with poor SRH among vegetable greenhouse farmers. A lower education level and never eating breakfast contributed to an increased likelihood of poor SRH in those with high PEI. The local government should be making great efforts to promote healthy behaviors and improve protection awareness.

## Introduction

Pesticides are widely used for the control of weeds and pests in order to expand profit from agricultural production. Studies to date have confirmed the positive association between pesticides exposure and health effects among farmers that range from acute poisoning to chronic hazards, such as mental disorder, brain tumors[1, 2], leukemia[3], renal disease[4], Parkinson’s disease[5] and depression[6]. Although a policy for banned highly toxic and restricted conventional pesticide use was implemented in China starting from the beginning of 2000, pesticide poisoning incidents are still reported. More than 200,000 people are killed due to pesticide poisoning every year in rural regions of developing countries, and most of them are caused by long-term, chronic pesticide exposure[7]. Individuals are exposed to pesticides by occupational or environmental exposures, and greenhouse workers are considered among the most vulnerable to pesticide exposure[8]. A study conducted in 189 intensive greenhouse workers and 91 healthy control subjects showed that chronic exposure to pesticides increased the risk of mild toxic effects, mainly including ocular and skin symptoms, and other unknown changes with long-term consequences[9]. The influence of chronic pesticide exposure on health becomes more important as the number of greenhouses workers increases due to China’s ranking first in world vegetable production[10].

Poor self-rated health (SRH) is a useful outcome measurement and more powerful predictor of comprehensive health status than the medical record as observed by clinical physicians and could predict functional disability, mortality and morbidity[11–15]. Links between poor SRH and human diseases, including health burden[16], microelement unbalance[17], mental health disorders[11], chronic diseases[11], and neurobehavioral disorders[18], have been reported. However, limited information is available about the relationship between SRH and pesticide exposure in greenhouse farmers. Some studies done in other countries have indicated the relevance of assessing pesticide exposure in related farms[19–22]. The aims of the current study were to evaluate the association between individual pesticide exposure intensity and SRH in greenhouse farmers.

## Materials and methods

### Study setting and participants

We conducted a population-based cross-sectional study from April to May in three consecutive years (2015, 2016, and 2017). Four large cooperative villages in Yinchuan City, the capital of Ningxia Province in Northwestern China, were chosen as the research sites. In the sample villages, most residents are engaged in vegetable greenhouse-related work. One non-repeated resident’s team (the smallest unit in the China rural area to facilitate management) was randomly selected in each village in each survey year. Then, all of greenhouse farmers in selected team who met the requirements were invited to participate in the investigation if they were agreeable. Participants enrolled criteria were: 1. worked in plastic vegetable sheds for at least one year; 2. lived at their current address for at least five years.

Information about pesticide exposures and SRH were collected via face-to-face interview by trained investigators using a questionnaire that assessed general demographic characteristics, medically diagnosed chronic diseases, pesticide spray behaviors, protection methods and hygiene habits, and SRH. Attempts to capture key missing data were supplemented at least twice by phone.

Because most participants in our study were busy with farming work at the time of our investigations, verbal consent from the participating farmers was obtained prior to the interview, which helped to facilitate management and improve investigation compliance and efficiency. The design for this study was approved by the Medical Ethics Committee of Ningxia Medical University (No.2014-090).

### Definitions and measurement

#### Pesticide exposure intensity

Pesticide exposure intensity (PEI) was estimated using a validated method from Dosemeci[23] and Lee[24]. The pesticide spray behavior item was added to this formula for the current study, based on the consideration that awareness of personal protection was low among vegetable farmers[25], and many farmers had unhealthy habits, such as chatting, eating and drinking water during the spray process, that would increase their exposure level. The PEI scores algorithm is as follows:

PEI = (mixing status + application method + spray behavior + whether the equipment was checked before spraying) × PPE × spills

The score for personal protective equipment (PPE) use was obtained by asking the following questions: “What are the protective measures you take when applying pesticides?” If “never” was selected, the participant was assigned to PPE-0. If one or more of the following options were selected, PPE-1 was assigned: masks, goggles, fabric or leather gloves, old clothes; if one or more of the following options were used, then PPE-2 was assigned: gas masks, rubber boots, or clean protective clothing; and if rubber gloves were used, then PPE-3 was assigned[23]. The PPE scores are shown in Table 1. A higher score indicates a lack of personal protection during the process of pesticide spraying.

**Table 1:** Specified scores for combinations of PPE use[23].

Type of PPE	Scores
PPE-0	1
PPE-1	0.8
PPE-2	0.7
PPE-3	0.6
PPE-1 & PPE-2	0.5
PPE-1 & PPE-3	0.4
PPE-2 & PPE-3	0.3
PPE-1 & PPE-2 & PPE-3	0.1

Mixing status was defined as two-levels, never and mixed, assigned to 0 and 9, respectively.

The pesticide spray application method was divided into three levels: hand spray, machine spray and mix spray; scores were 9, 3 and 6, respectively.

Behaviors during the spray process were divided into five categories: drinking water, eating, smoking, chatting, and none; scores were 3, 3, 2, 1 and 0, respectively.

Two options were set for whether the equipment was checked before spraying, check and never; scores were 0 and 2, respectively.

The spill score was derived from a single question “Did you change your clothes after spraying pesticides?”, and the corresponding score was obtained according to four response options (1 = “Immediately”; 1.2 = “At the end of the day”; 1.4 = “At will”; 1.8 = “Later in the week”).

Finally, we divided PEI into three groups (low, medium and high PEI groups) because of a skewed PEI distribution.

#### Self-rated health and covariates

SRH was measured using a single ten-point scale question: “What do you think of your health status compared with your peers?”. Respondents whose score ranged from 1 to 5 were referred to as “poor” SRH, and scores from 6 to 10 were referred to as “excellent” SRH, based on previous methods[26].

Chronic diseases were considered due to previous literature that showed strong associations with SRH[11] and were measured using a multiple-choice question: “Do you have the following chronic diseases that were diagnosed by a medical doctor/health professional?”. The options were as follows: “Hypertension”, “Coronary heart disease (CHD)”, “Hyperlipidemia”, “Stroke”, “Myocardial infarction”, “Heart failure”, “Coronary atherosclerosis” and “Others”. This question was then collapsed into a three-level categorical variable that indicated the number of chronic diseases (no disease, one chronic disease, and two or more diseases). Other potential confounders were smoking status, drinking status, breakfast frequency (What is the frequency of eating breakfast in your daily life?), investigation year, and family economic status (quartiles of family gross income minus family expenditure with Q1 representing the lowest family economic status and Q4 representing the highest family economic status).

### Statistical analyses

Chi-square tests were used to detect a difference in SRH by demographic characteristics, lifestyle characteristics, family economic status and PEI group.

A multivariable logistic regression model was used to examine the association between PEI and SRH. In the base model, only PEI was set as an independent variable. Demographic information, lifestyle variables, family economic status, number of chronic diseases and investigation year were adjusted singly or simultaneously in other models. The Odds Ratio (OR), 95% confidence intervals (CIs) and *P* value for the trend of poor SRH were calculated.

Interaction terms were defined as family economic status, education level and breakfast frequency multiplied by PEI. The interaction effect was evaluated by terms entered into the regression model. Significance was considered at *P*<0.05 for all of the tests. All analyses were performed in SPSS for Windows, version 24.0 (SPSS Inc., Chicago, IL, USA).

## Results

This study included 1366 participants (448, 460 and 458 participants were interviewed in 2015, 2016 and 2017, respectively). Of those who completed the survey, 725 were males and 641 were females. The average age was 46.84±10.27 years, ranging from 21 to 79 years. Hypertension and CHD accounted for a major proportion of chronic diseases in the current sample; 60 and 19 of them reported hypertension and CHD, respectively. Detailed sociodemographic characteristics of the participants are displayed in Table 2.

**Table 2:** Sociodemographic characteristics distribution by SRH among farmers of greenhouse vegetables, Yinchuan, China^a^.

Characteristics	Excellent SRH		Poor SRH		*P-*value^c^	*P-*value for trend^d^	OR (95% CIs)
	n	%	n	%			
Number of respondents^b^	1158	84.77	208	15.23			
Number of family members					0.147	0.152	
1 person	6	0.52	1	0.48	Ref.		Ref.
2 people	104	9.04	27	12.98	0.687		1.558(0.180–13.492)
3 people	206	17.91	27	12.98	0.827		0.786(0.091–6.783)
4 people and above	834	72.52	153	73.56	0.929		1.101(0.132–9.207)
Gender					0.718	-	
Male	617	53.28	108	51.92	Ref.		Ref.
Female	541	46.72	100	48.08	0.718		1.056(0.786–1.419)
Ethnicity					0.035	-	
Han	1016	87.74	193	92.79	Ref.		Reference
Hui	142	12.26	15	7.21	0.038		0.556(0.320–0.968)
Age group					0.387	0.393	
<20	57	4.92	10	4.81	Ref.		Ref.
20–30	298	25.73	47	22.60	0.778		0.678(0.309–1.487)
30–40	412	35.58	76	36.54	0.891		0.609(0.365–1.016)
40–50	279	24.09	46	22.12	0.869		0.712(0.443–1.147)
50–60	112	9.67	29	13.94	0.332		0.637(0.381–1.064)
Education level					<0.001	<0.001	
No formal school education	289	24.96	84	40.58	Ref.		Ref.
Primary school	363	31.35	73	35.27	0.039		0.692(0.488–0.981)
Junior high school	425	36.70	41	19.81	<0.001		0.332(0.222–0.496)
High school and above	81	6.99	9	4.35	0.010		0.382(0.184–0.793)
Marital status					0.401	-	
Unmarried	37	3.20	5	2.42	Ref.		Ref.
Married	1095	94.56	200	96.62	0.532		1.352(0.525–3.481)
Other	26	2.25	2	0.97	0.520		0.569(0.102–3.162)
Smoking status					0.080	-	
Everyday	410	35.41	80	38.65	Ref.		Ref.
Not everyday	15	1.30	7	3.38	0.066		2.392(0.945–6.053)
Former smoker (now quit)	51	4.40	11	5.31	0.777		1.105(0.552–2.213)
Never	682	58.89	109	52.66	0.212		0.819(0.599–1.121)
Drinking status					0.142	-	
At least 30 days ago	184	15.89	25	12.14	Ref.		Ref.
Within the last 30 days	258	22.28	39	18.93	0.697		1.113(0.651–1.903)
Never drink	716	61.83	142	68.93	0.103		1.460(0.926–2.301)
Breakfast					0.039	-	
Almost everyday	695	60.02	118	57.56	Ref.		Ref.
Occasionally	172	14.85	28	13.66	0.853		0.959(0.615–1.496)
Few	104	8.98	11	5.37	0.154		0.623(0.325–1.195)
Never	187	16.15	48	23.41	0.030		1.512(1.042–2.194)
Family economic status					<0.001	<0.001	
Q1	269	23.23	78	37.50	Ref.		Ref.
Q2	354	30.57	68	32.69	0.026		0.662(0.461–0.951)
Q3	265	22.88	40	19.23	<0.001		0.521(0.343–0.790)
Q4	270	23.32	22	10.58	<0.001		0.281(0.170–0.464)
Number of chronic diseases					0.333	0.338	
None	1093	94.39	191	91.83	Ref.		Ref.
One	47	4.06	13	6.25	0.155		1.583(0.840–2.981)
Two or more	18	1.55	4	1.92	0.667		1.272(0.426–3.798)
Investigation year					0.242	0.243	
2015	387	33.42	61	29.33	Ref.		Ref.
2016	393	33.94	67	32.21	0.681		1.082(0.744–1.572)
2017	378	32.64	80	38.46	0.111		1.343(0.935–1.928)
Pesticides exposure intensity					0.003	0.003	
Low level	362	34.48%	60	28.99%	Ref.		Ref.
Medium level	405	38.57%	67	32.37%	0.992		0.998(0.685–1.454)
High level	283	26.95%	80	38.65%	0.005		1.706(1.179–2.467)

Note

^a^ The sample size in the table may not total to 1366 due to missing data.

^b^ Percentage between SRH groups.

^c^The *P-*value for each group was derived from a Chi-square test. The trend *P-*value was derived from logistic regression.

^d^*P-*value for trend derived from logistic regression.

Of the participants, 208 (15.23%) reported poor health. A higher percentage of poor SRH were observed in high PEI (22.04%, 14.19% and 14.22% in the high, middle and low PEI groups, respectively). A significant distribution of poor SRH among ethnicity groups was determined, with Han higher than Hui (15.96% versus 9.55%). A low frequency of breakfast had a higher percentage of poor SRH than a regular breakfast habit. In the education groups, participants who had no formal school education had a high percentage (22.52%) of poor SRH, followed by primary school (16.74%), high school or above (10.00%) and junior high school (8.80%). In terms of family economic status, the percentage of poor SRH from Q1 to Q4 was 22.48%, 16.11%, 13.11% and 7.5%. In addition, a negative linear trend of poor SRH percentage was noted in education level and family economic status groups.

The association between SRH and PEI is shown in Table 3. Although a positive relationship between high PEI and poor SRH was observed, the association between SRH and medium PEI was insignificant. High PEI showed a steady association with poor SRH, with a 1.5-fold increased risk in the full model compared to low PEI.

**Table 3:** Association between PEI and poor SRH among greenhouse vegetable farmers, Yinchuan, China.

Model	Pesticides Exposure Intensity Level			*P-value* for Trend	*P-value* for interaction
	LEI	MEI	HEI		
Multivariable OR (95% CIs)					
Base Model^a^	Ref.	0.998(0.685–1.454)	1.706(1.179–2.467)	0.003	-
Base Model + Demographic information^b^	Ref.	0.984(0.671–1.442)	1.687(1.155–2.464)	0.005	0.039^c^
Base Model + Life-style factors^d^	Ref.	1.010(0.690–1.477)	1.640(1.125–2.391)	0.011	0.022^e^
Base Model + Family economic status	Ref.	1.030(0.705–1.506)	1.698(1.168–2.468)	0.006	0.126^f^
Base Model + Number of Chronic diseases	Ref.	0.993(0.681–1.447)	1.704(1.177–2.467)	0.003	-
Base Model + Investigate Year	Ref.	0.949(0.649–1.389)	1.637(1.128–2.376)	0.005	-
Full Model^g^	Ref.	0.927(0.626–1.374)	1.545(1.049–2.275)	0.016	-

Abbreviations: LEI, Low PEI; MEI, Medium PEI; HEI, High PEI.

^a^ Includes Pesticide Exposure Intensity level as an independent variable.

^b^Demographic information represents the Number of family members, Gender, Ethnic group, Age group, Education level, and Marital status.

^c^ *P*-value for the interaction between Education level and PEI.

^d^Lifestyle factors include smoking status, drinking status and breakfast frequency.

^e^ *P*-value for the interaction between Breakfast frequency and PEI.

^f^ *P*-value for the interaction between Family economic status and PEI.

^g^ Adjusted for all potential factors.

**Fig 1:**
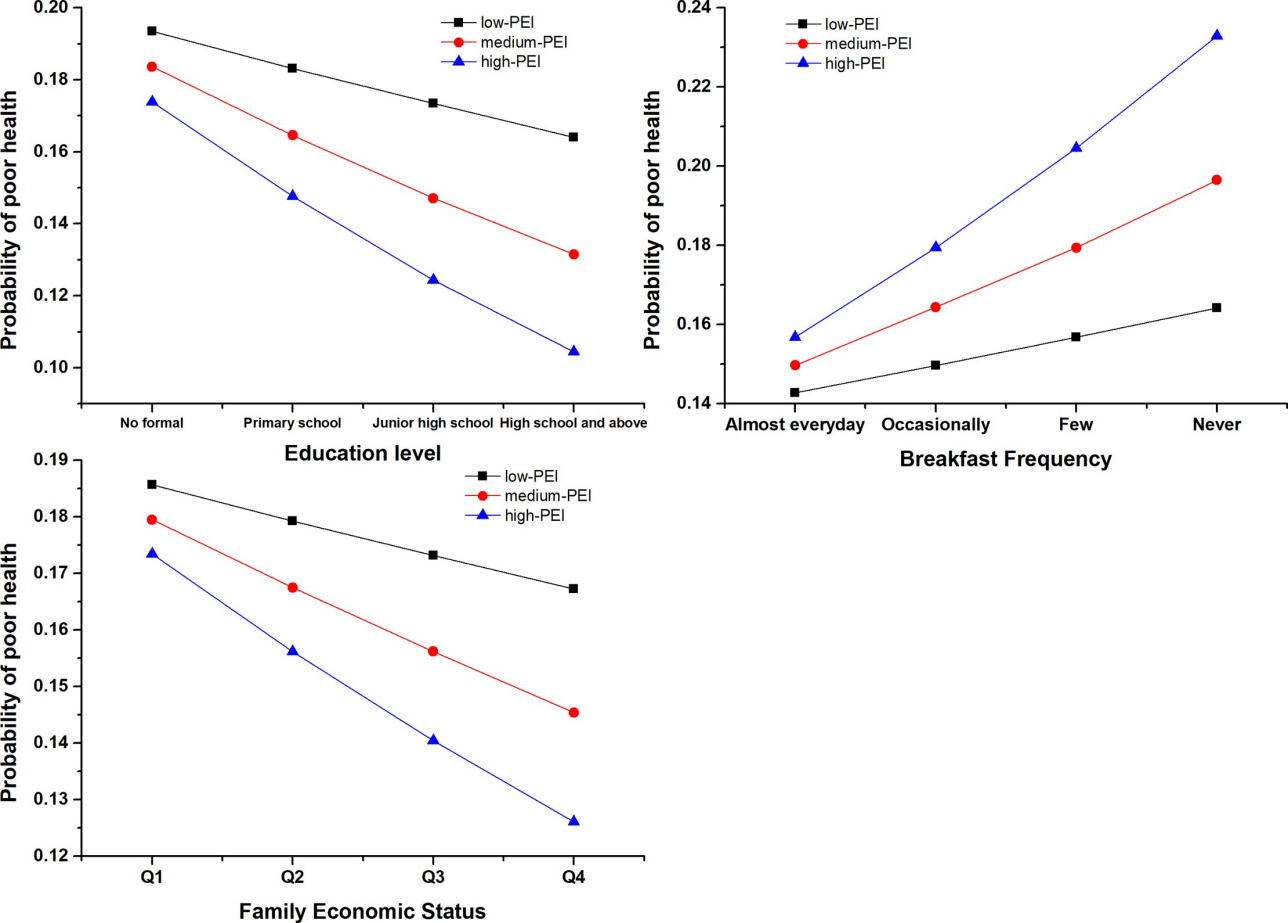
Interaction effects of PEI and education level, Breakfast frequency, and Family economic status among Chinese greenhouse vegetable farmers. Abbreviation: PEI: Pesticides Exposure Intensity (3-levels: Low, Medium, and High). Notes: The Y-axis is Probability of poor health calculated through logistic regression in which the dependent variable was SRH status (Poor = 2 and Excellent = 1) and the independent variables were each original variable used to derive the respective interaction variable. For example: in the left figure, the independent variables were education level and PEI; in the middle figure, the independent variables were Breakfast Frequency and PEI; in the right figure, the independent variables were Family economic status and PEI. Q1: First quartile of Family economic status; Q2: Second quartile of Family economic status; Q3: Third quartile of Family economic status; Q4: Fourth quartile of Family economic status.

In addition, interaction effects of education level (*p* = 0.039) and breakfast frequency (*p* = 0.022) with PEI were observed. Interaction plots are shown in Fig 1. The likelihood of poor SRH decreased with an increasing education level in the high PEI group, and a downward trend was obvious in the high PEI group. An interaction of PEI with breakfast habit showed a large slope, which suggested that the low frequency of eating breakfast would significantly increase the possibility of poor SRH in the high PEI group.

The insignificant association between poor SRH and medium PEI was verified in gender and ethnicity subgroups. Similarly, insignificant associations were also found between poor SRH and high PEI among females and the Hui ethnicity subgroups. However, the positive association between poor SRH and high PEI was confirmed in male and Han ethnicity subgroups, and the corresponding odds ratios were 1.954 (95% CI:1.124–3.395) and 1.581 (95% CI:1.056–2.366), respectively, from the full model. Details are presented in Table 4.

**Table 4:** Odds ratio for associations between PEI and Poor SRH in subgroups among greenhouse vegetable farmers, Yinchuan, China.

Model	Pesticides Exposure Intensity Level (OR, 95% CIs)								
	LEI	Male		Female		Han		Hui	
		MEI	HEI	MEI	HEI	MEI	HEI	MEI	HEI
Base Model^a^	Ref	1.314(0.762–2.268)	2.129(1.256–3.610)	0.772(0.456–1.308)	1.427(0.840–2.424)	1.017(0.688–1.501)	1.724(1.173–2.534)	0.810(0.188–3.478)	1.700(0.456–6.338)
Base Model + Demographic information^b^	Ref	1.258(0.724–2.185)	1.980(1.160–3.380)	0.757(0.443–1.293)	1.381(0.796–2.396)	0.946(0.657–1.448)	1.625(1.096–2.408)	0.818(0.182–3.672)	1.628(0.410–6.465)
Base Model + Lifestyle factors^c^	Ref	1.314(0.757–2.282)	2.073(1.213–3.543)	0.750(0.440–1.279)	1.330(0.774–2.285)	1.015(0.685–1504)	1.666(1.126–2.463)	0.691(0.155–3.079)	1.327(0.333–5.290)
Base Model + Family economic status	Ref	1.388(0.800–2.408)	2.149(1.260–3.663)	0.777(0.457–1.322)	1.410(0.826–2.406)	1.039(0.701–1.540)	1.741(1.180–2.570)	0.839(0.194–3.640)	1.632(0.431–6.171)
Base Model + Number of Chronic diseases	Ref	1.285(0.743–2.221)	2.137(1.258–3.629)	0.772(0.456–1.308)	1.426(0.839–2.424)	1.012(0.685–1.495)	1.722(1.171–2.532)	0.722(0.161–3.232)	1.607(0.427–6.055)
Base Model + Investigation Year	Ref	1.250(0.717–2.177)	2.058(1.208–3.505)	0.743(0.437–1.263)	1.362(0.798–2.326)	0.967(0.651–1.434)	1.663(1.128–2.451)	0.794(0.181–3.492)	1.652(0.418–6.529)
Full Model^e^	Ref	1.243(0.697–2.214)	1.954(1.124–3.395)	0.707(0.408–1.223)	1.274(0.723–2.245)	0.928(0.617–1.395)	1.581(1.056–2.366)	0.600(0.099–3.647)	0.908(0.183–4.493)

Abbreviations: LEI, Low Exposure Intensity; MEI, Medium Exposure Intensity; HEI, High Exposure Intensity.

^a^ Pesticides Exposure Intensity was the independent variable.

^b^Demographic information: Number of family members, Gender (excluded from Male and Female subgroup analysis), Ethnicity (excluded from Han and Hui ethnicity subgroup analysis), Age group, Education level, and Marital status.

^c^Lifestyle factors: smoking status, drinking status and breakfast frequency.

^e^Adjusted for all of the potential factors.

## Discussion

This study investigated the association of pesticide exposure intensity with self-rated health through validated assessment of pesticides exposure in the greenhouse vegetable farmers of western China. The results indicated a significant association between high PEI and poor SRH. The strength of association might be ensured by the fact that the results were observed in three consecutive surveys.

In the current study, SRH was used to efficiently reflect the health condition of greenhouse vegetable farmers. Although SRH was defined as a subjective perception that might be limited by education experience, age, gender and occupation characteristics[11, 27, 28], a previous study used it to reveal underlying disease burden[16] and it was viewed as an effective predictor of health status. Pesticide exposure was reported as a risk factor for health in previous studies[1, 2, 21, 29]. Our investigation is from a specific perspective that focused on vegetable greenhouse farmers because they might experience long-term low-level exposure to these toxic substances.

An insignificant association was observed between medium PEI and SRH; one plausible explanation is that this was a cross-sectional study that did not have enough power to capture a small effect of long-term low-dose exposure impact on health. From the full model, a 1.5-fold increased likelihood of poor SRH was observed in those with high PEI. The neural system and mental health are affected by pesticide exposure level and high susceptibility, and a relationship between specific pesticide exposures and a physiological biochemical index[18, 30, 31] or mental disorder[32, 33] has been demonstrated. This evidence might verify the association. Meanwhile, mental disorders showed a strong relationship with poor SRH and was also proven by previous research[34], and it is stated that individuals with high PEI had increased expression of negative emotions and lower self-perceived health.

A higher percentage of poor SRH was observed in the Han ethnicity compared to the Hui ethnicity, which might mean that religious faith played an important mediating role. All of the Hui people are hereditarily Islamic, but lack of faith in most Han people has been reported[35]. Religion would be positively associated with happiness according to results reported by Lelkes[36] and French[37]. A significant association between poor SRH and high PEI was determined in the male group but not in the female group. A supporting explanation is that men are usually the main physical laborers and, therefore, have more chances for pesticide exposure in the vegetable greenhouses than women.

A linear association between education level and SRH was estimated. Farmers who had a high education level usually had a good sense of self-protection behavior that could protect their health during the spray process. A significant relationship between breakfast frequency and poor SRH was identified, which is in line with results from Jieyu Chen[38], whose conclusion implied that this unhealthy lifestyle habit would affect health. Never eating breakfast was tightly associated with poor SRH in the current study. Financial status was positively associated with SRH, as was verified by a previous study[39]. Participants with high family economic status might have higher psychological satisfaction than participants from low-income families and be more likely to have good perceived health status due to their long-term superior social capital[40]. Another research study, conducted in a population-based survey, revealed that self-perceived economic satisfaction was associated with mortality among middle-aged and elderly adults [40]. However, the underlying mechanism needs a further multisite epidemiologic cohort to explore it.

An interaction effect of education level and PEI was shown as a rapidly descending trend in SRH from no formal education to high school among those in the high PEI group. A higher education level would help participants to gain knowledge of self-protection behaviors and select correct equipment when spraying pesticides. Another potential explanation might be that education had a mediating effect on adjusting mood and impacting body health[41]. For participants who never ate breakfast, there was a synergistic negative interaction with PEI to influence SRH. It could be possible that an irregular breakfast habit weakens the immune system and enhances the negative health effect of pesticide exposure.

Although SRH has been shown to be obviously advantageous for predicting health status compared to clinical examination, using it to assess health status might lead to bias. The PEI level was estimated by an indirect method, which might produce more bias than an objective measurement. Other limitations of this study should be noted. First, SRH status was measured at one time-point that did not consider the change or differences in pesticide exposure by season. Second, mediating effects from potential factors in PEI and SRH were not explored. Finally, the type of pesticides and their composition were not considered, which limits our ability to explain the mechanism.

This study is a reliable population-based study of the association between PEI and SRH in greenhouse vegetable farmers from western China. In conclusion, despite the above limitations, this study revealed a robust negative linear relationship between PEI and SRH from different models that adjusted for demographic, lifestyle and chronic disease. Meanwhile, the research provides visual confirmation of the interaction effects of education level and breakfast frequency with PEI. Low education and never eating breakfast could increase the odds of poor health with PEI, especially with high PEI. Apart from the causal pathways, the present multi-year cross-sectional survey implicates a needed effort by the local government to intervene in lifestyle and agricultural activities to reduce the risks associated with pesticides exposure among farmers.
